# Groundwater Quality in Sidi Slimane, Morocco

**DOI:** 10.5696/2156-9614-10.25.200309

**Published:** 2020-02-28

**Authors:** Nabil Darwesh, Ramzy S.M. Naser, Mohammed Al-Qawati, Shaker Raweh, Khadija El Kharrim, Driss Belghyti

**Affiliations:** 1 Laboratory of Agro-Physiology, Biotechnology, Environment and Quality, Faculty of Sciences, Ibn Tofail University, Kenitra, Morocco; 2 General Directorate of Environmental Health, Sana'a, Yemen; 3 Community faculty, Sana'a, Ministry of Technical Education and Vocational Training, Sana'a, Yemen

**Keywords:** drinking quality, groundwater, Sidi Slimane, Morocco

## Abstract

**Background.:**

Groundwater is an important source of drinking water for human and animal populations and therefore should be protected from pollution. The study area, Sidi Slimane, is in the western region of Morocco, which is a highly important agricultural area.

**Objectives.:**

The aim of the present study was to assess the suitability of groundwater for drinking in the Sidi Slimane region.

**Methods.:**

Twenty (20) samples were collected from different locations in the study area in 2015 in order to evaluate the quality of groundwater for human consumption. Collection, transportation and analysis of samples were performed according to the Standard Methods for the Examination of Water and Wastewater of the American Public Health Association (APHA), 2017. The major ions (cations and anions), ammonium (NH_4_^+^), pH and electrical conductivity (EC) of the groundwater samples were analyzed. Total dissolved solids (TDS) and total hardness (TH) parameters were calculated on the basis of obtained chemical data. ArcGIS program (version 10.4.1) was used in the preparation of topographic and hydrological maps of the study area.

**Results.:**

Groundwater showed high concentrations of most parameters of drinking water quality according to Moroccan and international standards. Groundwater was brackish and very hard. The results showed that sodium and chloride ions were the predominant ions. Salinity was present at low depths. The majority of groundwater samples in the study area (18 out of 20) were sodium chloride type, and only 2 samples were mixed calcium-magnesiumchlorine type.

**Discussion.:**

The obtained results were compared with the Moroccan standards for drinking water. The results show that 100%, 75%, 70%, 70%, 65% and 55% of groundwater samples exceeded the permissible limits for drinking water quality according to the Moroccan standard with regard to sodium, calcium, TDS, EC, chlorine, and nitrate, respectively.

**Conclusions.:**

Most of the groundwater samples in the study area showed poor drinking water quality. Groundwater quality must be protected by controlling the use of pesticides, fertilizers, manure, and harmful irrigation practices.

**Competing Interests.:**

The authors declare no competing financial interests

## Introduction

Globally, groundwater is a vital source of water supply for drinking, agriculture and the industrial sector. One-third of the world's population depends on groundwater, and about 40% of the world's food production uses groundwater.^1^,^2^

The increasing growth of the world economy, population and infrastructure has threatened an already stressed water supply. Assessment of water quality is more important than water quantity and provides important information for water management planning, particularly with regard to drinking water.^3^

Morocco is located in the far west of North Africa, its capital is Rabat, and its largest city is Casablanca. According to the 2014 General Population and Housing Moroccan census, the Moroccan population was 33,848,242.^4^ Morocco is characterized by an arid and semi-arid climate, with precipitation patterns that vary widely by season and place.

Groundwater in Morocco constitutes an important part of the hydraulic heritage of the country, however, it is subject to agricultural, industrial, and urban pollution.^5^ Morocco's water resources are limited. Morocco's renewable water resources are estimated at 29 billion m^3^ per year; in 1998 an average rate of 1044 m^3^ per capita/year was recorded, and by 2020, it is estimated to be only 786 m^3^ per capita/year.^6^

Morocco is currently experiencing water stress (less than 1000 m^3^/inhabitant/year) and is expected to experience a water shortage (less than 500 m^3^/inhabitant/year) after 2025.^7^ Water resources in the coastal area of the Gharb region are increasingly threatened by pollution caused by urban, agricultural, industrial and artisanal development. In addition to creating a growing water demand, recent development in the region has generated several polluted sites. The western region of Morocco plays a vital role in the country's agricultural production.

In the Sidi Slimane area in northwest Morocco, traditional methods are used for well digging; when water is at a depth of less than 10 m, manual drilling is employed with axes and shovels. At 30 m minimum depth, a drilling rig is widely used by local farmers. In addition to the use of natural fertilizers to fertilize soil (animal waste residues), farmers use synthetic fertilizers such as di-ammonium phosphate fertilizer complex granulated (DAP 18.46.0) and granulated compound fertilizer (NPK 10-20-20S + 6SO3).

Insecticides used in the area are generally chlorpyriphos. Ethyl, glyphosate and paraquat are widely used as herbicides.^8^ The most widely used wastewater disposal method in the region is the public sewer system (41.6%), followed by septic pits (37.7%), and 20.7% of the population uses abandoned wells or public sewer systems. The present study found that 84.2% of urban areas had public sewer systems compared to 4.8% of rural areas. Septic pits are used by 61.1% of rural residences, but only 10.5% of urban households.^9^ Abandoned wells are mainly used in rural areas, but only 2% are used in urban regions. Wheat, legumes, fodder, beetroot, sugar cane, citrus fruits and olives are grown in this area.^9^ Plants produce citrus fruits, including important crops such as oranges, lemons and limes. Improper practices and poor management of water irrigation and agrochemical inputs are the most important sources of groundwater deterioration.^7^ Moreover, consumption and use of poor quality water may lead to serious health problems.^10^

**Abbreviations**
*EC*: Electrical conductivity
*HCO_3_^−^*: Bicarbonates
*NH_4_^+^*: Ammonium
*NO_3_^−^*: Nitrates
*SO_4_^−2^*: Sulfates
*TDS*: Total dissolved solids
*TH*: Total hardness

Although sodium is necessary for human life, excessive doses may lead to deleterious health effects. Infants and young children are more vulnerable to the effects of sodium due to the immaturity of their kidneys.^11^ Calcium is also important for human health, particularly for the building of teeth and bones. The kidneys remove excessive amounts of calcium through urine. However, excessive calcium intake poses human health risks to those who are prone to milkalkali syndrome (the simultaneous existence of hypercalcemia, metabolic alkalosis, and renal insufficiency) and hypercalcemia.^12^

Magnesium is very important for human health as it participates in many vital processes in the human body, and a deficit of magnesium can lead to health problems.^13^ However, an increased intake of magnesium in drinking water, especially in the presence of high concentrations of sulfate, may cause diarrhea. The concentration of nitrates in ground and surface water is naturally low, but may become highly elevated due to leaching of cultivated land or contamination by waste of human or animal origin.^14^ An epidemiological study found that infants were at risk of methemoglobinemia when exposed to drinking water with nitrate levels >50 mg/L.^15^ Nitrates are very soluble in water, and when consumed by plants, they easily infiltrate the soil and gradually reach groundwater.^16^

The current study aims to determine the suitability of groundwater for drinking purposes in the Sidi Slimane area. As groundwater is an important source of drinking water for the local population, irrigation and farming, remedial solutions for securing its availability and ensuring its quality are strongly recommended.

## Methods

**Figure 1:**
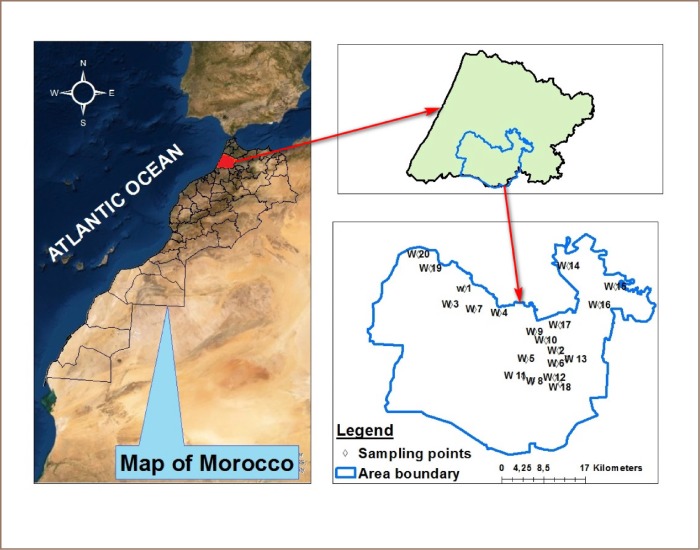
Location of Study Area

The Sidi Slimane area is part of the Gharb region in northwest Morocco and is located between longitudes 410,000 and 480,000 North and latitudes 380,000 and 430,000 East Universal Transverse Mercator *(Figure 1).* Sidi Slimane covers an area of 1517 km^2^. It is characterized by a semi-arid climate with high humidity coming from the west.

The average annual rainfall is 445 mm; the wettest month is December with an average of 77 mm rainfall. The dry period is relatively long and lasts from May to September. During this period, monthly rainfall does not exceed 5 mm. The average temperature varies between 10°C in January and 26°C in July and August. However, the average maximum temperature reaches 40°C in July and the average minimum is 1°C in December.

### Field and laboratory methods

A Global Positioning System (GPS) device was used to take the coordinates of wells in the study area. Groundwater samples were taken in May 2015 from 20 different locations in the Sidi Slimane region. Groundwater depth varied between 4 and 65 m. The samples were collected after pumping for 10 minutes. Clean and dry polyethylene bottles were used for sample collection, following standard procedures. The water samples were transported at low temperature (4°C) in portable coolers to the laboratory where tests were performed. The physicochemical parameters were analyzed according to the American Public Health Association (APHA) methods.^17^ Hydrogen ion concentration (pH) was measured using a pH meter (WTW Inolab). Electrical conductivity (EC) was measured using a conductivity meter (Thermo ORION 3 STAR); calcium (Ca^+2^) and magnesium (Mg^+2^) were determined by titration with ethylene diamine tetra-acetic acid; and sodium (Na^+^) and potassium (K^+^) were determined by a flame photometer (JENWAY PFP7).

Carbonates and bicarbonates(HCO_3_^−^) were determined by titration with a solution of 0.02 N sulfuric acids in the presence of phenolphthalein and bromocresol green as colored indicators. Chloride (C1^−^) was determined by the volumetric calibration with a standard solution of silver nitrate in the presence of potassium chromate as the detector. Sulfates (SO_4_^−2^) were measured using a spectrophotometer (V-1100). The determination of ammonium (NH_4_^+^) and nitrates (NO_3_^−^) was carried out by distillation using a distillation apparatus (Velp Scientifica UDK SER 148) in the presence of a reagent, magnesium oxide, and Devarda alloy (catalyst), respectively. During distillation, mineral nitrogen was fixed in boric acid as ammonium tetraborate and subsequently titrated with a sulfuric acid solution.

The ArcGIS program (version 10.4.1) was used in the preparation of topographic and hydrological maps of the study area. Minitab program (version 17) was used for analyzing the wells, according to the similarity of the chemical properties of groundwater in the study area. Total hardness (TH) of the groundwater was calculated using the formula given by Ragunath, 1987 *(Equation 1).*^18^

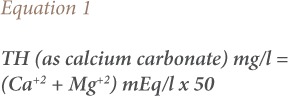
The pH values of the groundwater samples indicate that groundwater in the study area was slightly alkaline except for one sample which was slightly acidic. The accuracy of the chemical analysis of the major elements was verified by calculating the ionic balance between total cations and total anions for each water sample, using the ion charge balance equation or the ion balance error computation, as follows:

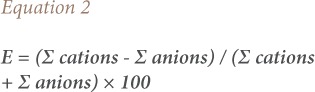
where, E is the error percent/reaction and Σ cations and Σ anions are the sums of the total cations and total anions expressed in mill equivalents per liter. The reaction (cationic and anionic balance) error (E) of all the groundwater samples was less than the accepted limit of ±5%, which supports the precision of the data.

Determination of the main ion sources of groundwater in the study area required a geochemical study.

To measure water quality, groundwater datasets were derived from various depths (4–65 m) during the study period and then evaluated.

## Results

The results and descriptive statistics of physicochemical parameters of groundwater samples collected from 20 sites in Sidi Slimane are displayed in Supplemental Material.

Through the ionic balance calculation in Table 1, the results showed that sodium and chloride were the predominant ions (Na^+^ was the most dominant ion, followed by Cl^−^). The order of the ions according to their availability in the groundwater of the study area was as follows: Na^+^> Cl^−^> Ca^+2^> HCO_3_^−^> Mg^+2^> NO_3_^−^> SO_4_^−2^>K^+^.

**Table 1:** Average Cation and Ionic Values of Groundwater in Study Area

**Cations mEq/L**	**Average**	**Anions mEq/L**	**Average**	**Error**
Ca^+2^	8.28	HCO_3_^−^	7.89	
Mg^+2^	5.74	NO_3_^−^	1.75	
Na^+^	31.73	CL^−^	31.56	3.33 %
K^+^	0.14	SO_4_^−2^	1.73	
Total	45.89	Total	42.93	

### Hydrochemical facies

**Figure 2:**
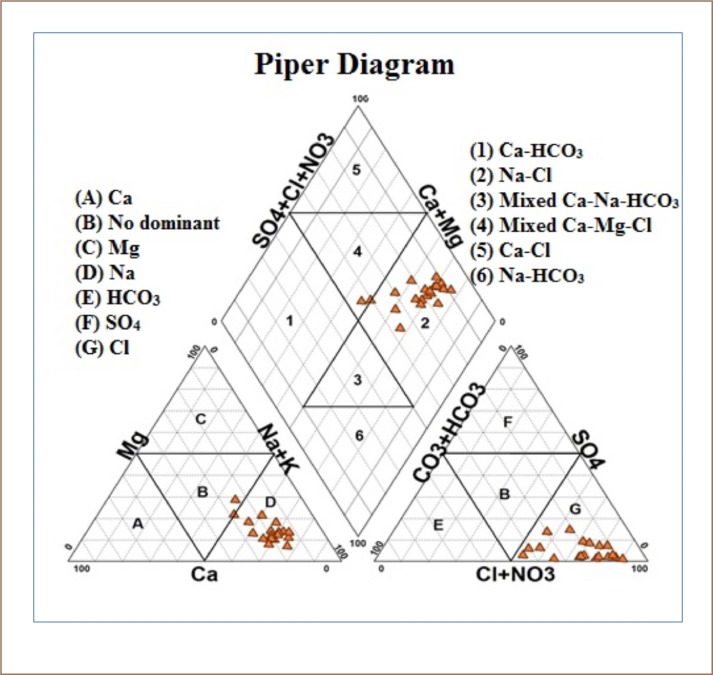
Piper diagram of all samples

In order to determine the geochemical nature of the groundwater in the study area, a Piper diagram was used to determine the hydrochemical facies. The Piper chart *(Figure 2)* shows that the majority of groundwater samples (18 out of 20) were a sodium-chloride type, and only 2 samples were mixed Ca-Mg-Cl type. Here, type refers to the chemical composition of the major elements of water. It means the dominance of cations and anions or the non-dominance by one of the cations. Sodium chloride type indicates that sodium is prevalent over the rest of the cations and chloride is prevalent over the other anions.

**Figure 3:**
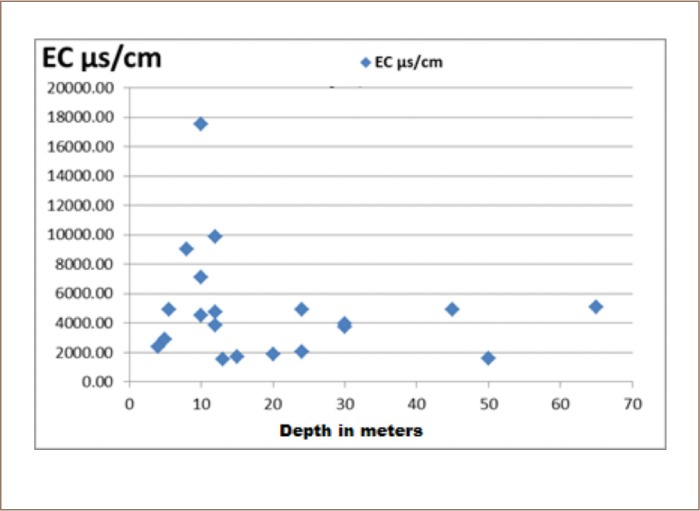
Relationship between EC of groundwater in μs/cm with well depths

The correlation coefficient is a measurement that determines the degree to which the movements of two variables are associated. Each cell in the table represents the relationship between two variables. The range of values for the correlation coefficient (denoted as r) is −1.0 to 1.0. A correlation of −1.0 indicates a perfectly negative correlation and a correlation of 1.0 indicates a perfect positive correlation. A value of zero indicates that there is no relationship between the two variables.^19^

The correlation matrices for 13 variables of groundwater quality parameters were prepared *(Table 2),* illustrating a very strong correlation between the following: TH-total dissolved solids (TDS), Ca^+2^-TH, Mg^+2^-EC, Mg^+2^-TH, Na^+^-EC, Na^+^- TH, Na^+^-Ca^+2^, Cl^−^-EC, Cl^−^-TH, Cl^−^-Na^+^ and TDS-EC. In addition, a strong correlation was found between the following: Ca^+2^-EC, Ca^+2^-TDS, Mg^+2^-Ca^+2^, Na^+^-Mg^+2^, K^+^ -EC, K^+^-TH, K^+^-Ca^+2^, K^+^-Na^+^, K^+^-Cl^−^, Cl^−^-Ca^+2^, Cl^−^-Mg^+2^, HCO_3_^−^-TH, and HCO_3_^−^- Ca^+2^. The pH exhibited a negative correlation coefficient (r) with most of the variables. The strong relationship between two parameters refers to the same source.

**Table 2:** Correlation Coefficient

	**EC**	**pH**	**TDS**	**TH**	**Ca^+2^**	**Mg^+2^**	**Na^+^**	**K^+^**	**Cl^−^**	**SO_4_^−2^**	**HCO_3_^−^**	**NO_3_^−^**	**NH_4_^+^**
EC	1												
pH	−0.069	1											
TDS	**1**	−0.069	1										
TH	**0.935**	−0.064	**0.935**	1									
Ca^+2^	**0.845**	−0.056	**0.845**	**0.941**	1								
Mg^+2^	**0.913**	−0.064	**0.913**	**0.941**	**0.77**	1							
Na^+^	**0.951**	−0.025	**0.951**	**0.952**	**0.916**	**0.875**	1						
K^+^	**0.772**	−0.036	**0.772**	**0.775**	**0.8**	**0.659**	**0.889**	1					
Cl^−^	**0.935**	−0.068	**0.935**	**0.934**	**0.899**	**0.859**	**0.99**	**0.89**	1				
SO_4_^−2^	0.23	−0.33	0.23	0.158	0.084	0.214	0.194	0.105	0.209	1			
HCO_3_^−^	**0.63**	−0.098	**0.63**	**0.733**	**0.716**	**0.662**	**0.652**	0.56	0.566	0.116	1		
NO_3_^−^	0.141	−0.174	0.141	0.28	0.385	0.142	0.272	0.3	0.211	−0.014	−0.559	1	
NH_4_^+^	−0.271	−0.094	−0.271	−0.252	−0.185	−0.289	−0.257	−0.183	−0.218	−0.344	−0.192	−0.15	1

Note: EC Values in μs/cm.

Abbreviation: TDS, total dissolved solids.

**Figure 4:**
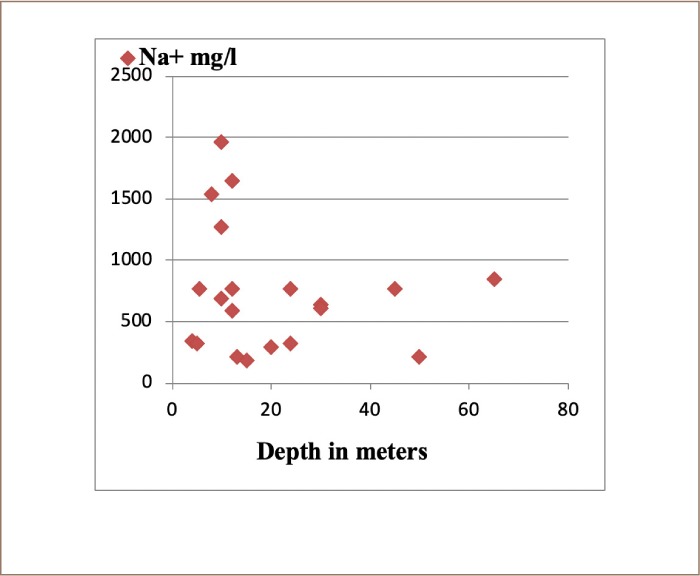
Relationship between concentration of Na^+^ in groundwater with well depth

**Figure 5:**
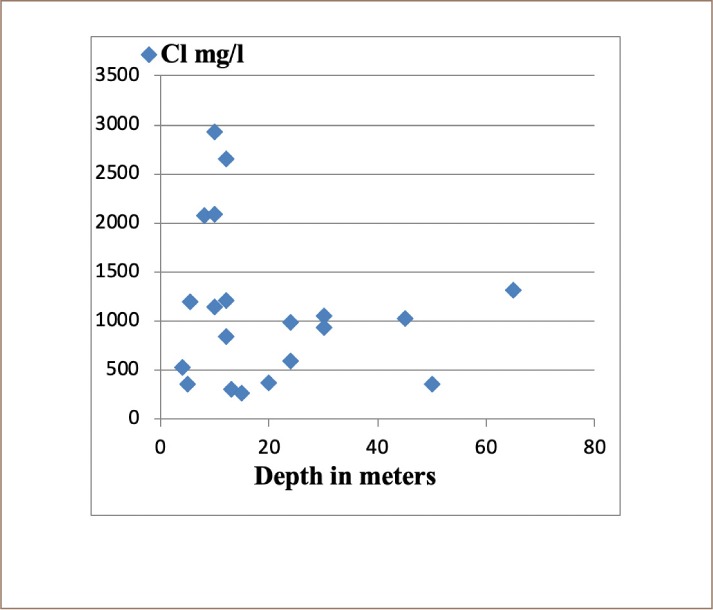
Relationship between concentration of Cl^−^ in groundwater with well depth

The chemical composition and hydrochemical facies of the groundwater samples indicate that sodium and chloride were dominant in groundwater and the correlation coefficient showed a strong correlation between these two parameters (r = 0.99) *(Figures 4 and 5)*. As shown in the values of the standard deviation of the data *(Supplemental Material),* it is clear that there was large variation in parameter values from one sample location to another.

According to Freeze and Cherry, the type of groundwater in Sidi Slimane is brackish, as shown in Table 3.^20^,^21^

**Table 3:** Nature of Groundwater in Sidi Slimane Based on Total Dissolved Solids Values

**TDS (mg/l)**	**Classification**	**Number of samples**	**% of samples**
<1000	Fresh water type	0	0.00 %
1000–10000	Brackish water type	20	100.00 %
Total		20	

The analytical results of the groundwater parameters in the study area were compared with the Moroccan standard.

Table 5 shows the number and percentage of wells that exceeded the permissible limits for various parameters for drinking water according to Moroccan standards. It is noted that 100%, 75%, 70%, 70%, 65% and 55% of groundwater samples exceeded the permissible limits for drinking water quality according to the Moroccan standards for Na^+^, Ca^+2^, TDS, EC, Cl^−^, and NO_3_, respectively.

**Figure 6:**
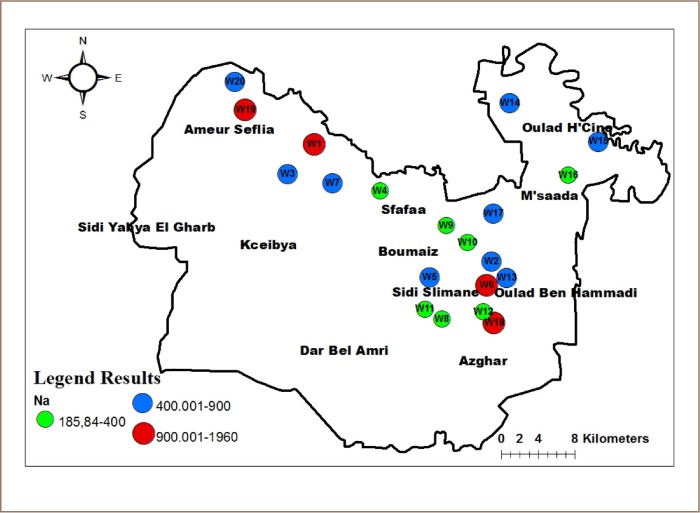
Distribution of sodium (Na) concentrations in the study area

Groundwater in the study area contained very high concentrations of sodium. Figure 6 shows that there were seven (7) wells with 186–400 mg/l of sodium, nine (9) wells with 400.01–900 mg/l, and four (4) wells with 900.01–1960 mg/l of sodium. The concentration of sodium ranged from 186 to 1959 mg/l, with an average of 738 mg/l. Wells 6 and 19 had very high sodium concentrations (1275 and 1959 mg/l), but very low nitrate concentrations (24 and 14 mg/l).

Concentrations of calcium in groundwater in the study area ranged from 56.8 to 290 mg/l, with an average of 167.5 mg/l. Seventy-five percent (75%) of samples had high concentrations of calcium compared with the Moroccan standards. Only three out of the 20 samples recorded high concentrations of magnesium compared to the Moroccan standards for drinking water.

**Figure 7:**
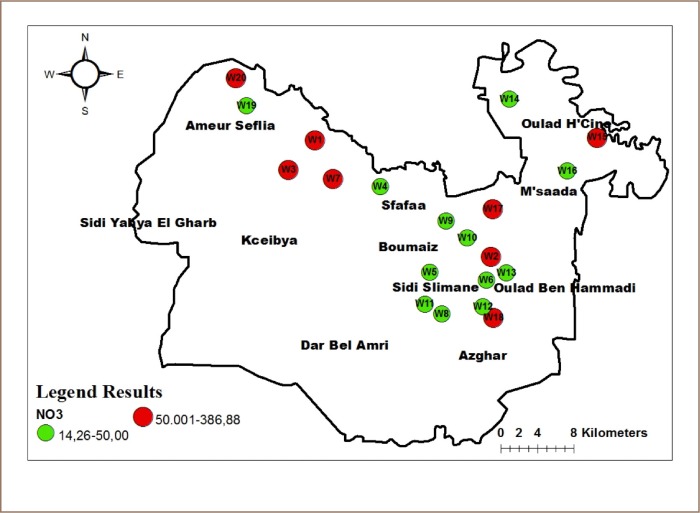
Distribution of nitrate concentrations in study area

Eleven (11) out of 20 groundwater samples had high concentrations of nitrates *(Figure 7),* which may be due to the use of agricultural fertilizers in the Sidi Suleiman region during agricultural irrigation. Concentrations of NO_3_^−^ in groundwater in the study area ranged from 14.26 to 386.88 with an average of 99.5 mg/l.

**Figure 8:**
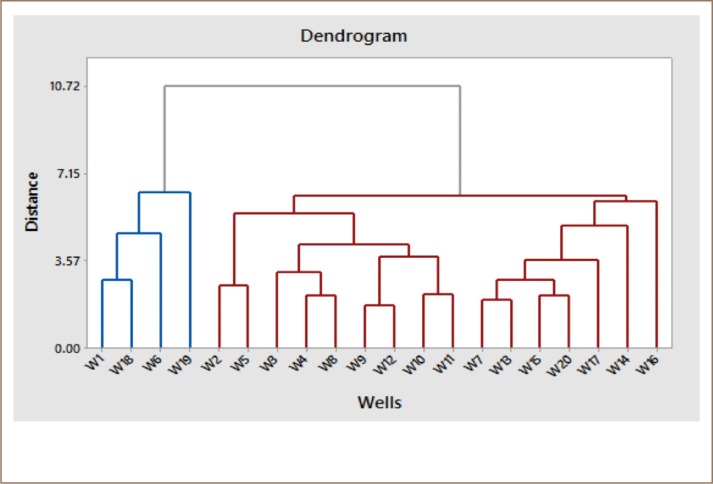
Dendrogram linking wells according to the similarity of the chemical properties of groundwater

Hierarchical cluster analysis refers to a statistical method for characterizing a set of data into groups according to their similarities.^22^ The dendrogram *(Figure 8)* shows the linkage of the 20 wells according to the similarity of the chemical properties of groundwater. Wells 1, 18, 6, and 19 are grouped in one category; wells 2, 5, 3, 4, 8, 9, 12, 10, and 11 are grouped together; and the rest of the wells fall into another category, indicating the convergence of groundwater quality of the wells in each category.

## Discussion

There was a high concentration of salinity in the groundwater of the study area. Figure 4 shows that high concentrations of electric conductivity (salinity) were present at low depths, giving an indication that the sources of salinity may be due to human activities. This does not prove that the rock layers are not involved in salt concentrations, especially rock salt (halite). It is also notable that salinity did not increase in wells with decreasing distance to the coast.

Due to the presence of high concentrations of sodium and chloride at depths close to the surface *(Figures 4 and 5),* they most likely have an anthropogenic source. This assumption confirms the ratio of chloride to sodium in the groundwater. This indicates that there is no intrusion of coastal (Atlantic) waters into the groundwater in the study area. It is clear from the results that there are high concentrations of sodium and chloride. These high concentrations in the groundwater of the study area indicate that water pollution is a result of human activities. Potassium ion concentrations in the groundwater of the study area were the lowest. The variation in the spatial distribution of potassium concentrations in groundwater of the Sidi Slimane region may be due to spatial variation in the use of agricultural fertilizers.

### Groundwater quality for human consumption

The quality of groundwater is as important as its quantity, as it is the main factor determining its suitability for drinking, domestic, agricultural and industrial purposes.^1^ Groundwater quality depends on several factors such as the level of weathering rocks, climate conditions of the region and the effect of human pollution (urban, industrial and agricultural activities).^23^

These aspects and their relational consequences create complex groundwater chemistry. This may reflect the impact of human activities in the study area on groundwater quality.

### Total hardness

According to Toumi *et al.*, all water samples in Sidi Slimane region are very hard as shown in Table 4.^24^

**Table 4:** Classification of Groundwater in Sidi Slimane Based on Total Hardness

**Total hardness as calcium carbonate (mg/1)**	**Water class**	**Number of wells**	**%**
<75	Soft	Nil	0.00 %
75–150	Moderately hard	Nil	0.00 %
>150–300	Hard	Nil	0.00 %
>300	Very hard	20	100.00 %

**Table 5:** Number and Percentage of Wells Exceeding Permissible Limits for Drinking Water Parameters According to Moroccan Standards in Study Area

	**Moroccan standard**	**Number of wells**	**Number of wells exceeding the permissible limits**	**% of wells above standard**
**pH**	6.5–9.5	20	0	0.00%
**EC _μs/cm_**	350–2700	20	14	70.00%
**TDS _mg/L_**	650–1500	20	14	70.00%
**TH _mg/l_**	500	20	14	70.00%
**Ca^+2^_mg/l_**	100	20	15	75.00%
**Mg^+2^_mg/l_**	100	20	3	15.00%
**Na^+^_mg/1_**	150	20	20	100.00%
**K^+^_mg/l_**	0–12	20	0	0.00%
**Cl^−^_mg/l_**	750	20	13	65.00%
**SO_4_^−2^_mg/l_**	250	20	0	0.00%
**HCO_3_^−^_mg/l_**	500	20	7	35.00%
**NO_3_^−^_mg/l_**	50	20	11	55.00%
**NH_4_^+^_mg/l_**	2	20	7	35.00%

^**^ indicates high concentrations of sulfate, in association with cations, such as magnesium, which may have a laxative effect on those unaccustomed to the water.

The results show that 13 out of 20 samples showed high chloride content. However, according to the World Health Organization, chloride toxicity has not been observed in humans except in special cases of impaired sodium chloride metabolism, e.g. in congestive heart failure.^25^ The high concentration of chloride in drinking water leads to an undesirable salty taste. The correlation between nitrate and sodium could reflect agricultural practices. The lowest concentration of sodium in groundwater exceeded the maximum allowable concentration of sodium in drinking water.

No high concentrations of sulfate were recorded in the study area, and therefore, water in the study area is not expected to cause laxative effects. The significant reason for hypomagnesemia is renal insufficiency related to a significantly decreased ability to excrete magnesium.^12^ Finally, high concentrations of nitrates in the groundwater of the study area puts the health of infants and elderly to risk, especially infants who drink milk in bottles contaminated with bacteria.

## Conclusions

It is clear from the chemical analysis of groundwater in the Sidi Slimane area that the majority of the groundwater in the study area is of poor drinking water quality, as it has high concentrations of most parameters of drinking water quality according to Moroccan and international standards. Groundwater in the study area is brackish and very hard. Sodium and chloride ions were the predominant ions. Salinity was present at low depths, indicating that the sources of salinity may be due to human activities. The majority of groundwater samples in the study area (18 out of 20) were a sodium chloride type, and only two samples were a mixed Ca-Mg-Cl type.

Water pollution is a growing concern in Morocco due to population growth, industrialization, and associated increased use of fertilizers and phytosanitary products in agriculture. Steps are being to be taken to address this environmental and public health concern, but the pace of progress is slow.

The present study has a couple of limitations, including limited sampling and lack of seasonal variation. Measures should be taken to regularly assess groundwater quality in order to mitigate pollution and protect water quality from further degradation.

## References

[1] Bhat MA, Wani SA, Singh VK, Sahoo J, Tomar D, Sanswal R (2018). An overview of the assessment of groundwater quality for irrigation. J Agric Sci Food Res.

[2] Li P, He S, Yang N, Xiang G (2018). Groundwater quality assessment for domestic and agricultural purposes in Yan'an City, northwest China: implications to sustainable groundwater quality management on the Loess Plateau. Environ Earth Sci [Internet].

[3] Naser R, EL Bakkali M, AL Nahmi F, Darwesh N, El Kharrim K, D (2017). Environmental contaminants and their impact on groundwater quality, in three area of Taiz, Yemen. Rev L'Entrep L'Innov [Internet].

[4] (2014). Recensement général de la population et de l'habitat 2014: note de présentation des premiers résultats [Internet].

[5] Belghyti D, Daifi H, Alemad A, Elkharrim K, Elmarkhi M, Souidi Y, Benelharkati F, Joti B, Elmoukrifi Z, Ibeda A, Azami-Idrissi Y, Baroud S, Elkhayyat F, Elrhaouat O, Sadeq S, Taboz Y, Sbai H, Naser R, Chigger H, Derwich N, Brebbia CA (2013). Groundwater management for sustainable production of drinking water quality in Maâmora. Water and society II [Internet].

[6] Bzioui M (2004). Rapport national 2004 sur les ressources en eau au Maroc [Internet]. Addis.

[7] El Khodrani N, Zouahri A, Arfaoui A, Iaaich H, El Oumlouki K, Yahyaoui A, Fekhaoui M (2016). Study of physico-chemical quality of groundwater in the rural commune of SFAFAA (Sidi Slimane Gharb, Morocco). J Mater Environ Sci [Internet].

[8] Maftouh I, Moussaif A, Elmzibri M, Elabbadi N, Mesfioui A (2017). Assessment of the main active molecules among the most used pesticides in the Gharb Region in Morocco. Int J Agric Innov Res [Internet].

[9] (2016). [Monograph of the Sidi Slimane region] [Monograph of the Sidi Slimane region] [Internet].

[10] Laferriere MJ, Minville J, Lavoie J, Payment P (1996). The hog industry and risks to human health. Bull Inf Heal Environ.

[11] (2003). Sodium in drinking-water: background document for development of WHO Guidelines for Drinking-water Quality [Internet].

[12] (2009). Calcium and magnesium in drinking-water: public health significance [Internet].

[13] Al Alawi AM, Majoni SW, Falhammar H (2018). Magnesium and human health: perspectives and research directions. Int J Endocrinol [Internet].

[14] El-Shaheny R, Fuchigami T, Yoshida S, Radwan MO, Nakayama M (2019). Complementary HPLC, in silico toxicity, and molecular docking studies for investigation of the potential influences of gastric acidity and nitrite content on paracetamol safety. Microchem J [Internet].

[15] Ward MH, Jones RR, Brender JD, de Kok TM, Weyer PJ, Nolan BT, Villanueva CM, van Breda SG (2018). Drinking water nitrate and human health: an updated review. Int J Environ Res Public Health [Internet].

[16] Alemad AK, Saadaoui H, said NA, Najy M, Daifi H, Yahya HS, Outhman A, Marc I, Aboubaker S, Belhaili I, Azami YI, El kharrim K, Belghyti D (2016). Note sur les causes de la dégradation de qualité des eaux souterraines de l'aquifère de sana' a -Yémen [Note about the causes of the deterioration of groundwater quality in aquifer of Sanaa -Yemen]. J Mater Environ Sci.

[17] Clesceri LS, Greenberg AH, Eaton AD (1998). Standard methods for the examination of water and wastewater.

[18] Ragunath HM (1987). Groundwater.

[19] Nickolas S What does it mean if the correlation coefficient is positive, negative, or zero?. Investopedia. [Internet].

[20] (2003). Total dissolved solids in drinking-water: background document for development of WHO Guidelines for Drinking-water Quality [Internet].

[21] Freeze RA, Cherry JA (1979). Groundwater.

[22] Shah B, Kansara B, Shankar J, Soni M, Bhimjiyani P, Bhanushali T, Shah M, Sircar A (2019). Reckoning of water quality for irrigation and drinking purposes in the konkan geothermal provinces, Maharashtra, India. Groundw Sustain Dev [Internet].

[23] Bhateria R, Jain D (2016). Water quality assessment of lake water: a review. Sustain Water Resour Manag [Internet].

[24] Toumi N, Hussein BHM, Rafrafi S, El kassas N (2015). Groundwater quality and hydrochemical properties of Al-Ula Region, Saudi Arabia. Environ Monit Assess [Internet].

[25] (2003). Chloride in drinking-water: background document for development WHO Guidelines for Drinking-water Quality [Internet].

