# Supplementary material for: Groundwater Quality in Sidi Slimane, Morocco
Source: J Health Pollut. 2020 Feb 28;10(25):200309. doi: 10.5696/2156-9614-10.25.200309 (PMC7058137; doi:10.5696/2156-9614-10.25.200309)
Supplement: Supplementary file 1 [file Darwesh_Supplemental.docx]

| **Supplemental Material**  **Chemical and Physical Analysis of Groundwater Samples in Study Area** | | | | | | | | | | | | | | |
| --- | --- | --- | --- | --- | --- | --- | --- | --- | --- | --- | --- | --- | --- | --- |
| **Number** | **Depth**  **(m)** | **pH** | **EC µs/cm** | **TDS** | **TH** | **Ca^+2^** | **Mg^+2^** | **Na^+^** | **K^+^** | **Cl^-^** | **SO_4_^-2^** | **HCO_3_^-^** | **NO_3_^-^** | **NH_4_^+^** |
| W1 | 12 | 7.50 | 9890.00 | 6428.50 | 1415.95 | 290.00 | 168.00 | 1646.60 | 9.40 | 2656.11 | 79.68 | 591.70 | 170.50 | 2.34 |
| W2 | 5.5 | 7.31 | 4910.00 | 3191.50 | 752.47 | 202.40 | 60.00 | 770.30 | 6.20 | 1196.40 | 223.44 | 591.70 | 152.50 | 1.44 |
| W3 | 30 | 7.01 | 3720.00 | 2418.00 | 594.42 | 130.00 | 65.52 | 637.30 | 4.30 | 1055.10 | 140.60 | 346.50 | 122.80 | 2.90 |
| W4 | 24 | 7.19 | 2050.00 | 1332.50 | 323.45 | 85.60 | 26.64 | 318.80 | 3.90 | 587.90 | 72.50 | 145.20 | 41.50 | 2.70 |
| W5 | 12 | 6.74 | 4750.00 | 3087.50 | 708.00 | 164.80 | 72.00 | 770.30 | 7.02 | 1210.60 | 192.50 | 486.80 | 52.10 | 1.44 |
| W6 | 10 | 7.06 | 7130.00 | 4634.50 | 851.28 | 239.20 | 61.68 | 1274.90 | 10.90 | 2090.20 | 34.20 | 356.20 | 23.70 | 1.98 |
| W7 | 10 | 7.09 | 4520.00 | 2938.00 | 740.73 | 169.20 | 77.28 | 690.50 | 8.20 | 1149.50 | 25.48 | 484.30 | 146.90 | 2.16 |
| W8 | 50 | 7.15 | 1590.00 | 1033.50 | 309.86 | 57.60 | 40.32 | 212.50 | 3.90 | 358.60 | 113.30 | 208.60 | 18.60 | 1.44 |
| W9 | 20 | 7.85 | 1880.00 | 1222.00 | 315.94 | 56.80 | 42.28 | 292.10 | 3.90 | 372.04 | 56.60 | 418.50 | 18.60 | 1.44 |
| W10 | 5 | 7.04 | 2900.00 | 1885.00 | 707.90 | 160.80 | 74.40 | 318.80 | 2.70 | 356.40 | 56.60 | 488.00 | 28.50 | 0.90 |
| W11 | 15 | 7.31 | 1700.00 | 1105.00 | 464.79 | 86.00 | 60.72 | 185.84 | 2.34 | 264.83 | 20.50 | 400.16 | 23.56 | 1.98 |
| W12 | 13 | 7.63 | 1540.00 | 1001.00 | 352.98 | 81.20 | 36.48 | 212.52 | 3.12 | 301.75 | 113.30 | 311.10 | 66.34 | 2.16 |
| W13 | 12 | 7.14 | 3850.00 | 2502.50 | 659.04 | 182.00 | 49.68 | 584.20 | 3.90 | 844.19 | 34.20 | 500.20 | 96.72 | 2.88 |
| W14 | 65 | 7.71 | 5110.00 | 3321.50 | 781.20 | 204.80 | 65.52 | 849.85 | 5.85 | 1317.76 | 43.32 | 368.44 | 31.00 | 2.52 |
| W15 | 45 | 7.36 | 4900.00 | 3185.00 | 779.96 | 195.20 | 71.04 | 770.27 | 5.07 | 1028.79 | 52.44 | 556.32 | 141.63 | 1.62 |
| W16 | 4 | 7.47 | 2410.00 | 1566.50 | 486.69 | 130.00 | 39.36 | 345.23 | 5.07 | 519.72 | 13.68 | 478.24 | 28.52 | 7.02 |
| W17 | 24 | 7.38 | 4900.00 | 3185.00 | 755.35 | 199.20 | 62.64 | 770.27 | 5.85 | 986.90 | 50.20 | 653.92 | 386.88 | 1.98 |
| W18 | 8 | 7.60 | 9020.00 | 5863.00 | 1115.45 | 250.00 | 119.28 | 1540.31 | 11.70 | 2074.62 | 79.80 | 799.10 | 252.96 | 0.90 |
| W19 | 10 | 7.17 | 17550.00 | 11407.50 | 1513.78 | 289.20 | 192.24 | 1959.37 | 9.75 | 2928.75 | 141.36 | 677.10 | 14.26 | 1.08 |
| W20 | 30 | 7.62 | 3980.00 | 2587.00 | 591.65 | 175.20 | 37.44 | 610.88 | 5.85 | 927.26 | 36.48 | 370.88 | 171.74 | 1.62 |
| Minimum | 4 | 6.74 | 1540 | 1001 | 309.9 | 56.8 | 26.64 | 186 | 2.340 | 265 | 13.7 | 145.2 | 14.26 | 0.90 |
| Maximum | 65 | 7.85 | 17550 | 11408 | 1513.8 | 290.0 | 192.24 | 1959 | 11.7 | 2929 | 223.4 | 799.1 | 386.88 | 7.02 |
| Average | 20 | 7.32 | 4915 | 3195 | 711.0 | 167.5 | 71.13 | 738 | 5.95 | 1111 | 79.0 | 461.6 | 99.5 | 2.13 |
| St. dv. | - | 0.28 | 3772 | 2452 | 330.5 | 70.5 | 42.58 | 507 | 2.74 | 776 | 58.0 | 158.5 | 96.6 | 1.30 |
| Results in mg/l except for pH and EC.  Abbreviations: St. dv., standard deviation; W, wells; TDS, total dissolved solids; EC, electrical conductivity. | | | | | | | | | | | | | | |
